# Multiple human papillomavirus infections are highly prevalent in the anal canal of human immunodeficiency virus-positive men who have sex with men

**DOI:** 10.1186/s12879-014-0671-4

**Published:** 2014-12-16

**Authors:** Rocío Méndez-Martínez, Norma E Rivera-Martínez, Brenda Crabtree-Ramírez, Juan G Sierra-Madero, Yanink Caro-Vega, Silvia C Galván, David Cantú de León, Alejandro García-Carrancá

**Affiliations:** División de Investigación, Instituto Nacional de Cancerología, Secretaría de Salud (SSA), México D.F., Mexico; Instituto Nacional de Ciencias Médicas y Nutrición “Salvador Zubirán,” SSA, México D.F, Mexico; Dirección de Investigación Clínica, Instituto Nacional de Cancerología, Secretaría de Salud (SSA), México D.F., Mexico; Instituto de Investigaciones Biomédicas, Universidad Nacional Autónoma de México, México D.F., Mexico; Unidad de Investigación Biomédica en Cáncer, Instituto de Investigaciones Biomédicas, Universidad Nacional Autónoma de México and Instituto Nacional de Cancerología, SSA, México D.F., Mexico; Laboratory of Virus & Cancer Instituto Nacional de Cancerología, Av. San Fernando No. 22, Colonia Sección XVI, México D.F., 14080 Tlalpan Mexico

**Keywords:** HIV, HPV, MSM, Type 16, Variants, Anal cancer

## Abstract

**Background:**

Anal cancer has become one of the most common non-AIDS-defined tumors among Human Immunodeficiency Virus-positive (HIV+) individuals, and a rise in its incidence among HIV+ Men who have Sex with Men (MSM) has been shown, despite the introduction of Highly Active Anti-Retroviral Therapy (HAART). Human Papillomavirus (HPV) infections are highly prevalent among HIV+ MSM and recent studies have shown high rates of HPV-associated anal intraepithelial neoplasia (AIN) and anal cancer among this population.

**Methods:**

In the present study we determined the prevalence and nature of HPV co-infections in the anal canal of 324 HIV+ MSM attending a high specialty medical center in Mexico City, DNA extraction and amplification with generic primers for HPV was performed, followed by detection of specific types and co-infections with INNO-Lipa, and identification of variants by amplification and sequencing of the E6 and LCR region of HPV 16.

**Results:**

We found a very high prevalence of HPV infections among this cohort (86%), with more than one fourth of them (28%) positive for type 16. Among HPV16-positive patients, European variants were the most prevalent, followed by Asian-American ones. Among these individuals (HPV-16+), we identified co-infections with other 21 HPV types namely; 11, 51, 52, 6, 66, 68, 74, 18, 45, 35, 26, 44, 70, 53, 54, 82, 31, 33, 56, 58, 59.

**Conclusions:**

HIV+ MSM show a very high rate of HPV infections in the anal canal and those with type 16 exhibited a multiplicity of associated types. This study emphasizes the need for an early detection of HPV infections among HIV+ MSM in order to establish its utility to prevent anal neoplasia and cancer.

**Electronic supplementary material:**

The online version of this article (doi:10.1186/s12879-014-0671-4) contains supplementary material, which is available to authorized users.

## Background

HPV are well known for their association with cervical cancer [1], and have also been shown to play a role in the pathogenesis of distinct squamous cell cancers, including anal [2],[3], penile [4],[5], oropharyngeal [6],[7] vulvar, and conjunctival [8] cancers. Concurrent infection with HIV may facilitate or accelerate the pathological consequences of HPV infections. Persistent HPV infections are very frequent among HIV+ MSM [9]. Anal HPV infections, which contribute to the development of anal warts and anal cancer, are very common among MSM, especially HIV+ individuals [10] and HPV-associated ano-genital malignancies occur particularly in patients with Acquired Immunodeficiency Syndrome (AIDS) [11]. HIV+ MSM are at 37-fold greater risk of invasive anal cancer. Likewise, the incidence of anal cancer and anal intraepithelial neoplasia (AIN), the potential precursor lesion of squamous cell carcinoma of the anus is very high among HIV+ MSM [12] and a definite increasing trend in the incidence of anal cancer has been shown despite the use of Highly Active Antiretroviral Therapy (HAART) [13].

Infections with HPV represent the most common sexually transmitted disease worldwide. HPV is a double-stranded DNA virus, and 160 different types of HPV officially described have been found at mucosal or cutaneous sites, each with a specific tissue tropism. At least 30 of them have been identified with high predilection for the ano-genital tract. Specific types, such as 6 and 11, are classified as low-risk (LR) types because they have been found to be associated only with benign lesions. In contrast, high-risk (HR) types, including most notably 16 and 18, have been found to be associated with both low- and high-grade anal squamous intraepithelial lesions (ASIL) as well as the majority of cervical and anal cancers [14],[15].

Risk factors for the presence of anal HPV include the presence of anal warts and a history of receptive anal intercourse, HIV infection, and a low CD4 cell count [16]. In a Spanish cohort HR and LR HPV types were very prevalent in the anus of HIV+ MSM (83% and 72.7%, respectively), with type 16 being the most common one. Concurrent infection with several HPV types was also common among HIV+ MSM (58.5%) [17]. In addition, HIV+ men have higher rates of HPV anal infection and higher levels of HPV type 16 than HIV- men [16],[18].

In a recent study in immunocompetent heterosexual men, the incidence of overall ano-genital HPV infections was 24.8% [19].

The anal and cervical epithelia possess similar embryonic origins. Histologically, columnar epithelium with a transition zone (with increased metaplastic activity) and a more differentiated squamous epithelium are observed. The anal and cervical epithelia are infected by the same types of HPV, producing similar manifestations that range from *condyloma* to squamous intraepithelial lesions (SILs) and cancer.

The fact that multiple concurrent infections with HPV constitute an associated morbidity among patients infected with HIV has only recently been recognized [20].

In Mexico, there have been few reports describing the prevalence of anal HPV among HIV+ MSM. In 2002, we studied a group of 31 HIV+ MSM from Mexico City and detected HPV in the anal canal of 74.2% of the cases; 67.7% were positive for HR HPV types (hcII test B: 16/18/31/33/35/39/45/51/52/56/58/59/68), and 64.5% were positive for LR HPV types (hcII test A: 6/11/42/43/44) [21].

In this study, we determined the prevalence of HPV types infecting the anal canal of HIV+ MSM and characterized the type 16 variants. In addition, we characterized the HPV types associated with HPV type 16 in the anal canals of these patients.

## Methods

### Samples

We analyzed 324 anal exudates from HIV+ MSM patients attending the HIV Clinic at the Instituto Nacional de Ciencias Médicas y Nutrición “Salvador Zubirán” (INCMNSZ) in Mexico City. Patients over 18 years old, who presented to care between August and December 2008, were invited to participate. Samples were obtained with a cytobrush and inserted into a tube collector containing PreservCyt. The study was approved by the Ethics Committees of INCMNSZ SSA, and the Instituto Nacional de Cancerología, SSA. Written informed consent was obtained from all of the participants.

### Data collection

Socio-demographic, clinical and sexual behavior information was collected using a self-applied written questionnaire, with assistance of one of the researchers (NR-M) who aid participants if needed. Clinical information was verified and completed with information available in the clinic records.

### Detection and typing of HPV

Extraction and purification of DNA was performed with the Genomics Wizard kit (PROMEGA). Polymerase chain reaction (PCR) was performed using the MY09/11 primers, which detect a fragment from the L1 gene. Positive samples were evaluated in a second PCR with specific primers to detect the E6 gene of HPV type 16 or a fragment form the LCR of HPV type 18. Negative samples underwent a second PCR using the GP5+/6+ primers, which detect a shorter fragment from the L1 gene and those positive underwent PCR reactions to detect type 16 E6 gene or an LCR fragment form type 18. Finally, all negative samples were subjected to a final PCR to identify a fragment from the β-globin gene to rule out problems of DNA quality or integrity. HPV type 16+ samples were subjected to a PCR to identify variants within E6 and the long control region (LCR) with specific primers. The PCR products were sequenced using the Big Dye terminator kit and an AB Applied Biosystems Prism 3100. For the identification of the other viral types present in the samples, we used the INNO-LiPA HPV Genotyping Extra kit (INNOGENETICS), which detects 28 different HR types of HPV (16, 18, 26, 31, 33, 35, 39, 45, 51, 52, 53, 56, 58, 59, 66, 68, 73 and 82) as well as a number of low-risk HPV genotypes (6, 11, 40, 43, 44, 54, 70) and the additional types 69, 71, and 74.

### Statistical analyzes

The statistical analyzes were performed by using the Kruskall-wallis test for continuous variables and chi-square test and Fisher-exact test were done to compare proportions of categorical variables.

## Results

A total of 324 MSM patients attending the HIV/AIDS Clinic at the INCMNSZ between August and December 2008 were invited and agreed to participate in this study. The majority was young adults (39.1, SD = 9.4) years old, educated and economically active (79.2% had more than 9 years of education, and 77.1% were employed at entry).

The mean duration of HIV infection since diagnosis was 83 (SD = 66.4) months. Approximately 58.4% of the participants had received a diagnosis of AIDS at some point during the course of their HIV disease. Three hundred two (92.4%) of 324 patients were being treated with HAART; 85.1% of the patients exhibited a T lymphocyte CD4 count above 200 cells/μl (mean = 442 cells/ ml), and 75% exhibited <400 copies/ml of HIV-1 RNA. A significant association was found between presence of HPV and age. HPV- patients were older than those that were HPV+ (Table 1).

**Table 1:** **Demographic and Clinic characteristics of patients included**

Variable	Total N = 324	HPV+ N = 279	HPV- N = 45	p-value
Age	39 (33–45)	38 (32–44)	42 (35–49)	0.015
Education year	12 (10–16)	12 (10–18)	12 (10–16)	0.777
Employed	206 (64)	179 (65)	27 (59)	0.637
CD (cells/mL)	414 (267–592)	409 (267–578)	459 (287–699)	0.267
Viral Suppression	268 (83)	228 (82)	41 (91)	0.127
Time since diagnoses (years)	6.22 (2.99-9.9)	6.12 (2.80-9.47)	7.80 (4.29-12.0)	0.077
Time since treatment (years)	2.76 (0.5-6.2)	2.60 (0.50-6.00)	3.77 (0.96-7.39)	0.212
Regiment of *HAART Initiation				
PI	99 (31)	88 (32)	11 (24)	0.718
NNRTI	175 (54)	148 (55)	27 (61)	
Other	39 (12)	33 (12)	6 (13)	

Notes: Median and Interquartile range reported for continuous variable. Number of patients and percentage reported for categorical variables. Viral suppression defined as HIV-RNA <400 copies/mL.

*Information available only for 313 patients.

Regimens: PI Protease Inhibitor, NNRTI Non-nucleoside Reverse transcriptase Inhibitors, Other.

The total prevalence of HPV in the anal canal of this population was very high (86%; 279/324). Of the HPV+ samples, 27.5% were positive for type 16 (77/279), 8.6% for type 18 (24/279), 36% for both types (101/279) and 64% for other HPV types (178/279). Finally, only 45 samples were found to be HPV- (13.6%) (Table 2).

**Table 2:** **Summary of HPV**

TOTAL (n)	HPV (n)	HPV16/HPV18* (N)	HPV16-HPV18 - (N)	HPV16+ (n)	HPV18+ (n)	HPV-
100% (324)	86.1% (279)	27.5% (89)	58.6% (190)	23.8% (77)**	7.4% (24)	13.9% (45)

Notes: *Patients were positive for HPV16 and/or HPV18. **There were 12 patients that were doubly infected by HPV type 16 and 18.

**Figure 1:**
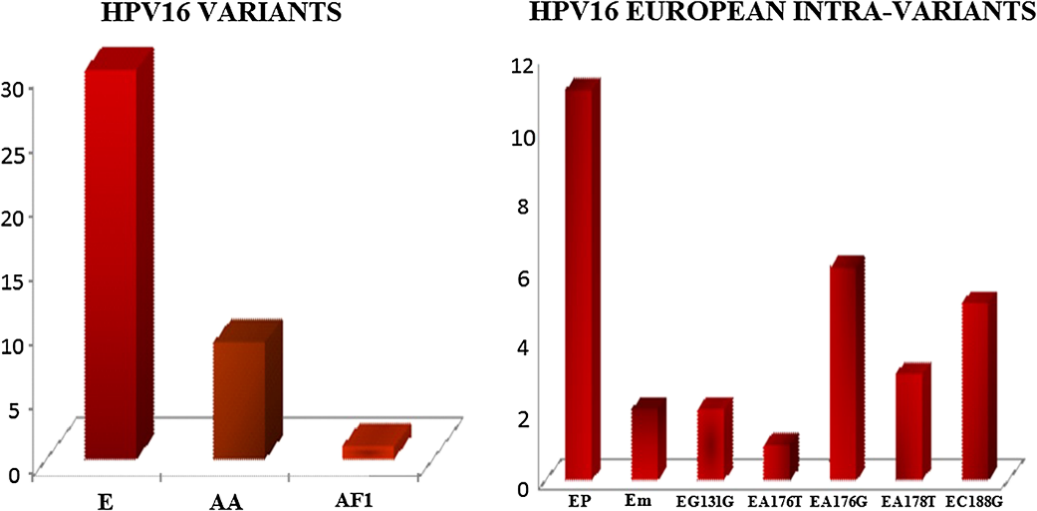
**European variants of HPV-16 are highly prevalent among HIV+ MSM.** A total of 39 clinical samples, were sequenced across the E6 (nt 104–559) and LCR (nt 7480–7843) regions to determine variant and intra-variant types. Variants and intra-variants were classified according to Yamada et al. [23].

In order to try to characterize HPV type 16 variants present in this population, and due to limited resources, 39 HPV16+ samples were subjected to further analysis by sequencing the LCR and E6 regions. Among this sub-population, three variants of HPV type 16 were identified. The most prevalent variants (70%) were the European variants (E), followed by Asian American (AA) variants (22.5%) and African (Af) variants (2.5%). Within the European variants, we identified 7 different subclasses: E-P in 11 samples, E-m in 2 samples, E-G131G in 2 samples, E-A176T in one sample, E-A176G in 6 samples, E-A178T in 3 samples, and E-C188G in 5 samples. Concerning the Asian American variants, we identified 2 subclasses: AAa in 6 samples and AAc in 3 samples. Finally, an Af variant was identified in one sample. All of these results are summarized in Figure 1. The fact that the majority of type HPV type 16 variants present in this population were of the European lineage could suggest a reduced risk to develop anal lesions and cancer for individuals carrying them, as compared to individual that carry Asian-American variants, has been shown for cervical cancer [22].

**Figure 2:**
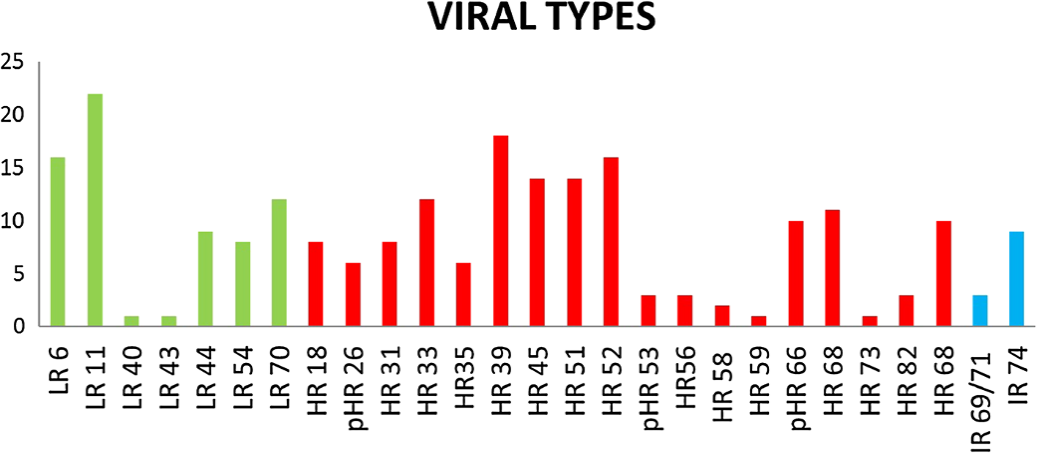
**High and wide prevalence of high risk HPVs co-infecting the anal canal of HPV16-HIV+ MSM.** Graphical representation of different viral types found in the sample analyzed, as described in Methods. Colors indicated associated risk; Low (Green Rectangle), High (Red Rectangle), or indeterminate (Blue Rectangle).

In addition, we analyzed which other types of HPV were present in this sub-population of 39 samples of HPV type 16+ individuals. The most frequent co-infecting HPV genotypes were 6, 11, 44 and 70 among the low-risk types; 16, 18, 52, 26, 31, 33, 35, 39, 45, 51, 52, 53, 56, 58, and 59 among the high-risk types; and 66 (probable high risk), 69/71 and 74, which are currently considered indeterminate risk (see Figure 2).

**Figure 3:**
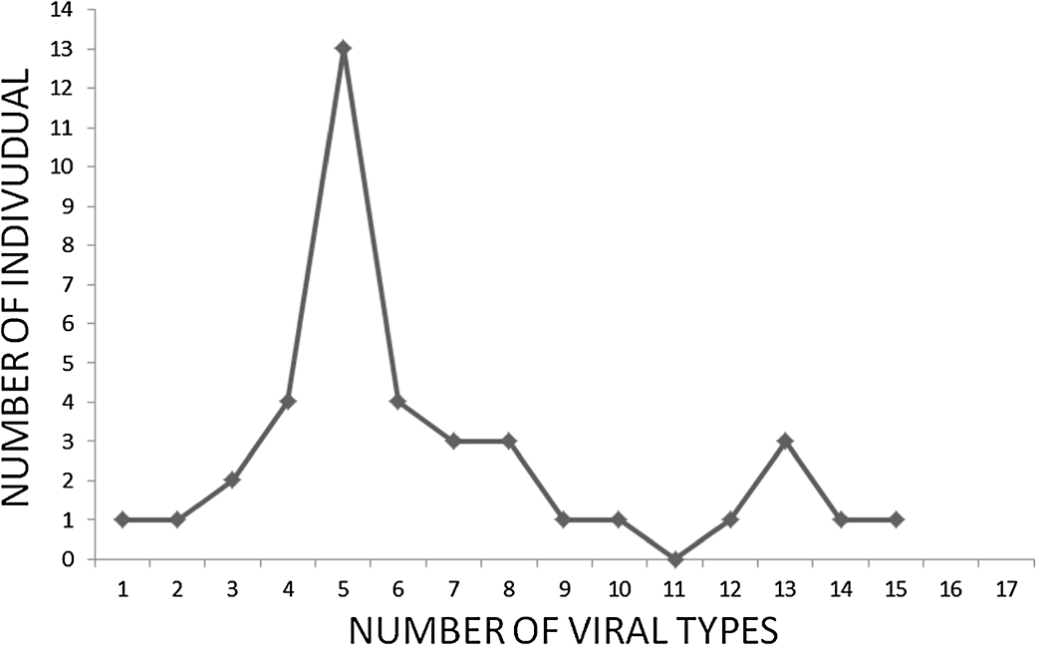
**More than half of the patients have between 4 and 6 different viral types.** The number of different viral types among individuals was determined as described in Methods.

**Figure 4:**
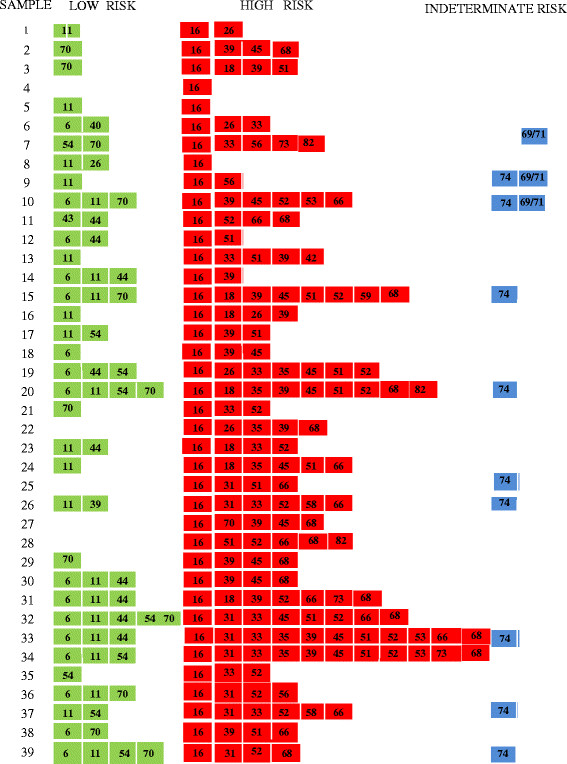
**Multiplicity of HPV types coinfecting the anal canal of HIV+ MSM, additional HPV type found among HPV type 16-positive HIV+ MSM.** 39 HPV type 16 positive cases were analyzed by INNO-LiPA to determine additional viral genotypes present in each sample.

The distribution of the number of different HPV types in this sub-population is shown in Figure 3. Two peaks, one of 11 individuals with 5 different viral types and the other with 3 individuals with 13 different types were seen. We observed that only 5% of the 39 HPV type 16+ samples analyzed were singly or double infected, whereas 95% of the type 16+ samples exhibited multiple HPV infections. Among multiply infected samples, 5% (2/39) had 3 viral types, 13% (5/39) had 4 viral types, 28.5% (11/39) had 5 viral types, 10.25% (4/39) had 6 viral types, and 7.7% (3/39) had 7, 8 or 13 different viral types. A detailed analysis of the different types of HPV present in this population is shown in Figure 4.

We performed a statistical analysis to compare clinical variables among HPV type 16/18+ and HPV- patients and found that age, viral suppression and time since diagnosis were significantly different among them. HPV- patients were older, have more HIV viral suppression and more time since diagnosis than HPV16/18+ patients (see Table 3).

**Table 3:** **Demographic and Clinic characteristics of patients between HPV negatives and patients with HPV16 and/or HPV18**

Variable	HPV16/HPV18 N = 89*	HPV N = 45	p-value
Age	38 (32–44)	42 (35-49	0.029
Education years	12 (10–16)	12 (11–16)	0.803
Employed	57 (65)	27 (59)	0.780
CD4(cells/mL)	354 (244–506)	459 (287–699)	0.069
Viral Suppression	68 (76)	41 (91)	0.044
Time since diagnoses (years)	4.30 (1.63-8.55)	7.80 (4.29-12.0)	0.006
Time since treatment (years)	2.11 (0.21-5.44)	3.77 (0.96-7.39)	0.090
Regimen of ** HAART Initiation			
PI	36 (43)	11 (24)	0.136
NNRTI	42 (50)	27 (61)	
Other	6 (7)	6 (13)	

Notes: Median and Interquartile range reported for continuous variable. Number of patients and percentage reported for categorical variables. Viral suppression defined as HIV-RNA <400 copies/mL.

*89 patients distributed as: 65 patients with HPV16, 12 patients with HPV18 and 12 patients with HPV16 and HPV18.

**Information available for 84 patients of HPV16/HPV18 and 45 or HPV-group.

Regimens: PI Protease Inhibitor, NNRTI Non-Nucleoside Reverse Transcriptase Inhibitor, Other.

Further studies comparing the prevalence of HPV in the oral cavity among these patients will be performed with a follow-up that could address the prevalence of HPV and anal lesions among this cohort of HIV+ MSM.

## Discussion and conclusions

HPV has been extensively studied for its role in cervical cancer development, but its association with anal cancer is less well defined. This study was conducted among HIV+ MSM attending a specialty medical center in Mexico City. We observed a high prevalence of HPV infections in the anal region, confirming previous reports [17],[18]. HR HPV types were most prevalent among a subgroup of HPV16+ patients, similar to what has been shown in previous studies of patients in Spain, Denmark and Sweden [17],[24]. The fact that HPV16 was the most prevalent is in agreement with previous reports. To our knowledge this is the first report on the presence of variants of HPV16 among HIV+ MSM.HPV16 was found in 23.8% of our patients, similar to what has been reported previously [25]. It is known that infection with HIV increases the risk of acquisition of HPV and suggests that persistence of HPV may also be increased [24].

The high prevalence of anal HPV, particularly type 16 could considerably increase the possibility of developing anal cancer among this population. Our results suggest that the early detection and treatment of lesions in the anal epithelium of these individuals may prevent anal cancer, one of the few non–AIDS-defining malignancies. Priority should be given to the identification and treatment of anal lesions in MSM with HIV/AIDS [26].

In Mexico, few data on this type of cancer, and even fewer with respect to the HIV+ population, have been obtained. Here, we describe the prevalence of different HPV types co-infecting individuals with HPV type 16, and we identify the type 16 variants in the anal canal of a population of HIV+ MSM.

## Authors’ original submitted files for images

Below are the links to the authors’ original submitted files for images. Authors’ original file for figure 1 Authors’ original file for figure 2 Authors’ original file for figure 3 Authors’ original file for figure 4

## References

[1] Bosch FX, de Sanjose’ S (2003). Chapter 1: human papillomavirus and cervical cancer–burden and assessment of causality. J Natl Cancer Inst Monogr.

[2] Pfister H (1996). The role of human papillomavirus in anogenital cancer. Obstet Gynecol Clin North Am.

[3] Welton ML, Sharkey FE, Kahlenberg MS (2004). The etiology and epidemiology of anal cancer. Surg Oncol Clin N Am.

[4] Dillner J, von Krogh G, Horenblas S, Meijer CJ (2000). Etiology of squamous cell carcinoma of the penis. Scand J Urol Nephrol Suppl.

[5] Aboulafia DM, Gibbons R (2001). Penile cancer and human papilloma virus (HPV) in a human immunodeficiency virus (HIV)-infected patient. Cancer Invest.

[6] Mork J, Lie AK, Glattre E, Hallmans G, Jellum E, Koskela P, Koskela P, Moller B, Pukkala E, Schiller JT, Youngman L, Lehtinen M, Dillner J (2001). Human papillomavirus infection as a risk factor for squamous cell carcinoma of the head and neck. N Engl J Med.

[7] Herrero R, Castellsague X, Pawlita M, Lissowska J, Kee F, Balaram P, Rajkumar T, Sridhar H, Rose B, Pintos J, Fernández L, Idris A, Sanchez JM, Nieto A, Talamini R, Alessandra T, Bosch XF, Ulrich R, Snijders PJF, Meijer CJL, Viscidi R, Munóz N, Franceschi S (2003). Human papillomavirus and oral cancer: the International Agency for Research on Cancer multicenter study. J Natl Cancer Inst.

[8] Gillison ML, Shah KV (2003). Chapter 9: role of mucosal human papillomavirus in nongenital cancers. J Natl Cancer Inst Monogr.

[9] Kreutera A, Wieland U (2009). Human papillomavirus-associated diseases in HIV-infected men who have sex with men. Curr Opin Infect Dis.

[10] Gao L, Zhou F, Li X, Yang Y, Ruan Y, Jin Q (2010). Anal HPV infection in HIV-positive men who have sex with men from China. PLoS One.

[11] Frisch M, Biggar RJ, Goedert JJ (2000). Human papillomavirus associated cancers in patients with human immunodeficiency virus infection and acquired immunodeficiency syndrome. J Natl Cancer Inst.

[12] Palefsky J (2009). Human papillomavirus-related tumors in HIV. Curr Opin HIV AIDS.

[13] de Pokomandy A, Rouleau D, Ghattas G, Trottier H, Vézina S, Coté P, Macleod J, Allaire G, Hadjeres R, Franco EL, Coutlée F (2014). HAART and progression to high-grade anal intraepithelial neoplasia in men who have sex with men and are infected with HIV. Clin Infect Dis.

[14] Palefsky JM, Gonzales J, Greenblatt RM (1990). Anal intraepithelial neoplasia and anal papillomavirus infection among homosexual males with group IV HIV disease. JAMA.

[15] Biggar RJ, Marital MM (1996). Status in relation to Kaposi’s Sarcoma, non-Hodgkin’s lymphoma, and anal cancer in the pre-AIDS era. J Acquir Immunodefic Syndr Hum Retrovirol.

[16] Critchlow CW, Hawes SE, Kuypers JM, Goldbaum GM, Holmes KK, Surawicz CM, Kiviat NB (1998). Effect of HIV infection on the natural history of anal human papillomavirus infection. AIDS.

[17] Torres M, González E, del Romero J, Pompeyo V, Ocampo A, Rodríguez-Fortúnez P, Másia M, Blanco JR, Portilla J, Rodríguez C, Hernández-Novoa B, del Amo J, Ortiz M (2013). Anal human papillomavirus genotype distribution in HIV-infected men who have sex with men by geographic origin, age, and cytological status in a Spanish cohort. J Clin Microbiol.

[18] Machalek D, Poynten M, Jin F, Fairley CK, Farnsworth A, Garland SM, Hillman R, Petoumenos K, Roberts J, Tabrizi SN, Templeton D, Grulich AE (2012). Anal human papillomavirus infection and associated neoplastic lesions in men who have sex with men: a systematic review and meta-analysis. Lancet Oncol.

[19] Nyitray A, Nielson CM, Harris RB, Flores R, Abrahamsen M, Dunne EF, Giuliano AR (2008). Prevalence of and risk factors for anal human papillomavirus infection in heterosexual men. J Infect Dis.

[20] Czelusta A, Yen-Moore A, Van der Straten M, Carrasco D, Stephen K (2000). An overview of sexually transmitted diseases. Part III. Sexually transmitted diseases in HIV-infected patients. J Am Acad Dermatol.

[21] Rabkin CS, Biggar RJ, Melbye M, Curtis RE (1992). Second primary cancers following anal and cervical carcinoma: evidence of shared etiologic factors. Am J Epidemiol.

[22] Villanueva RP, Diaz PP, Guido JM, Rangel PA, Sotelo RH, Garcia-Carranca A (2002). Prevalencia de virus papiloma humano de alto riesgo en el epitelio anal de hombres VIH positivos. Bioquímica.

[23] Yamada T, Manos MM, Peto J, Greer CE, Muñoz N, Bosch FX, Wheeler CM: Human papillomavirus type 16 sequence variation in cervical cancers: a worldwide perspective. *J Virol* 1997, 2463-2472*.* , 10.1128/jvi.71.3.2463-2472.1997 PMC191357 9032384

[24] Donà MG, Palmara G, Di Carlo A, Latini A, Vacaturo A, Benevolo M, Pimpinelli F, Giglio A, Moretto D, Impara G, Giuliani M (2012). Prevalence, genotype diversity and determinants of anal HPV infection in HIV-uninfected men having sex with men. J Clin Virol.

[25] Berumen J, Ordoñez RM, Lazcano E, Salmeron J, Galvan SC, Estrada RA, Yunes E, Garcia-Carranca A, Gonzalez-Lira G, Madrigal-de laCampa A (2001). Asian-American variants of human papillomavirus 16 and risk for cervical cancer: a case–control study. JNCI.

[26] Critchlow CW, Surawicz CM, Holmes KK, Kuypers J, Daling JR, Hawes SE, Goldbaum GM, Sayer J, Hurt C, Dunphy C (1995). Prospective study of high grade anal squamous intraepithelial neoplasia in a cohort of homosexual men: influence of HIV infection, immunosuppression and human papillomavirus infection. AIDS.

