# Supplementary figures and images for: Multiple human papillomavirus infections are highly prevalent in the anal canal of human immunodeficiency virus-positive men who have sex with men
Source: BMC Infect Dis. 2014 Dec 16;14:671. doi: 10.1186/s12879-014-0671-4 (PMC4272559; doi:10.1186/s12879-014-0671-4)

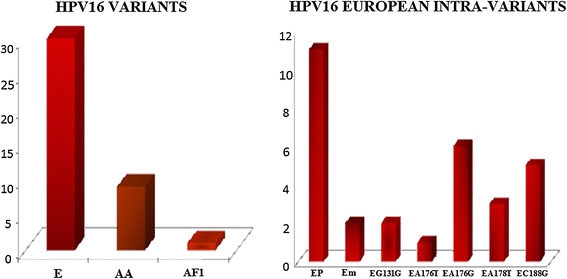

Supplement: Supplementary file 1 — Authors’ original file for figure 1 [file 12879_2014_671_MOESM1_ESM.gif]

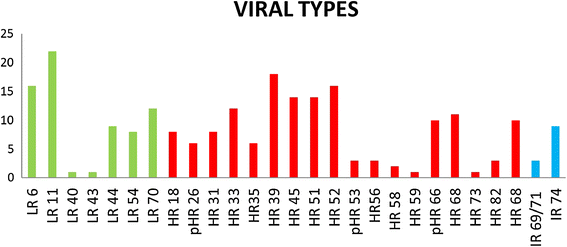

Supplement: Supplementary file 2 — Authors’ original file for figure 2 [file 12879_2014_671_MOESM2_ESM.gif]

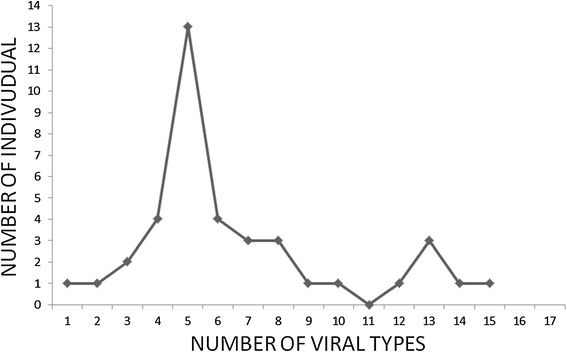

Supplement: Supplementary file 3 — Authors’ original file for figure 3 [file 12879_2014_671_MOESM3_ESM.gif]

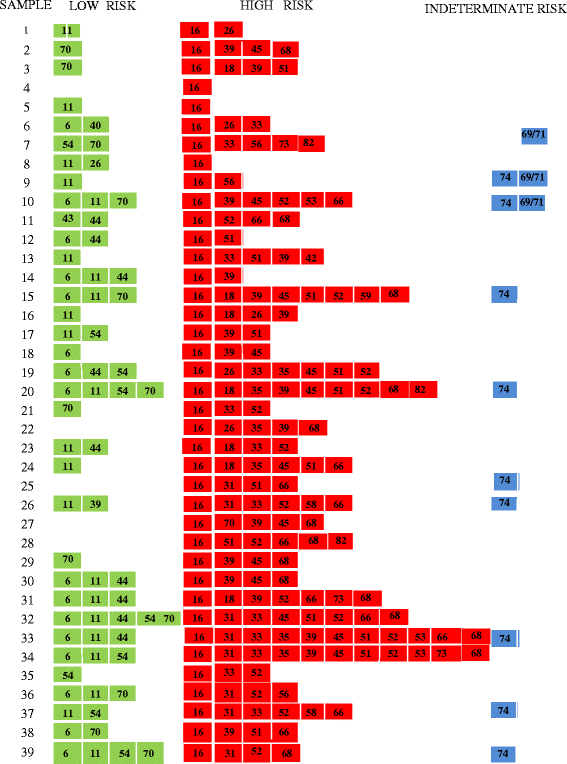

Supplement: Supplementary file 4 — Authors’ original file for figure 4 [file 12879_2014_671_MOESM4_ESM.gif]
